# Impact of Socio-Economic Factors on Nutrition Efficiency: An Application of Data Envelopment Analysis

**DOI:** 10.3389/fnut.2022.859789

**Published:** 2022-04-25

**Authors:** Mohammad Reza Pakravan-Charvadeh, Cornelia Butler Flora, Ali Emrouznejad

**Affiliations:** ^1^Department of Agricultural Economics and Rural Development, Lorestan University, Khorramabad, Iran; ^2^Department of Sociology, Kansas State University, Manhattan, KS, United States; ^3^Department of Sociology, Iowa State University, Ames, IA, United States; ^4^Surrey Business School, Faculty of Arts and Social Sciences, University of Surrey, Guildford, United Kingdom

**Keywords:** data envelopment (DEA), efficiency, optimization, socio-economic disparity, nutrient intake, urban-rural areas

## Abstract

**Background:**

Paying particular attention to sustainable food consumption in low-income households is essential for increasing human health. Due to the growing population globally, this concept will likely become more serious soon.

**Methods:**

Following the importance of optimizing food consumption for sustainability, in this study, a novel methodology is introduced for calculating nutrient intake efficiency and determining choices of food in different locations. The impact of socio-economic factors on nutrition efficiency is assessed. Data Envelopment Analysis (DEA) as a well-known linear programming (LP) and a Tobit model are used to achieve the goals. Household Consumption and Expenditure Surveys (HCESs) of 30,000 rural and urban Iranian households in all provinces in 2016 are analyzed. A Nutrient Efficiency Map (NEM) of Iran was depicted by GIS software.

**Results:**

The results showed that many townships had nutrient efficiency scores of less than 70%. Northeast townships had the lowest scores, with an efficiency score of less than 50%. Overall, townships have lower efficiency in the North (seaside cities), East (desert cities), and North East (isolated cities) when compared with other areas.

**Conclusion:**

Therefore, it is suggestible that the government should modify the support policies and the protection packages based on social, geographical, and cultural status.

## Introduction

The availability of healthy foods, nowadays, is a global concern. It depends on diverse, sustainable food systems. Therefore, paying particular attention to sustainable food available for consumption in low-income households is essential for ensuring a healthy population (1). This can be achieved through choosing the best combination of food groups (2). Sustainable consumption of foods prevents or mitigates the loss of resources and thus translates into a considerable improvement in food security (3).

Using Food Consumption Optimization (FCO), to choose the best combination of food ingredients, can lead to a decrease in household expenditure while nutrition is optimized. Households become more efficient in allocating limited food expenditures, leading to increased nutrient efficiency. The FCO reduces nutritionally inefficient food consumption that is at least partly caused by inaccurate or insufficient knowledge. Nutritional knowledge can be a critical policy component to combat malnutrition and to improve diet. Knowledge gaps exist in different regions and for different commodities, and these discrepancies may lead to a qualitative change in households’ diet from healthy to unhealthy status. Increasing attention to food diversity and nutrient characteristics has two substantial benefits in improving the quality of life: first, it allows households to avoid unhealthy diets and thus second, it reduces the adjustable risk for non-communicable disease-related mortality worldwide (4). As Khan (5) shows, adopting the mortality-related axiom of biological stress in poverty measurement leads to a more accurate measure of poverty headcount ratio. The first benefit of increasing diverse nutrient intake is decreasing the impacts of poverty. The second benefit of avoiding less nutritious food is monetary savings. Methods of food group selection, preparation, and combination affect human health and reduce the prevalence of such diseases as diabetes, heart attacks, and strokes, among other non-communicable diseases (6).

Data Envelopment Analysis (DEA), a well-known linear programming (LP) application that was initially proposed by Charnes et al. (7), is a method for measuring the relative performance of homogenous Decision-Making Units (DMUs) with multiple inputs and outputs (8). The Tobit model is a technique for determining associated factors with calculated efficiency. This model, first reported by Tobin (9), has been widely used to determine effective factors on efficiency (10). Tobit regression is frequently used by economists to analyze discontinuous dependent variables and to analyze dependent variables that subject to known upper or lower bounds (11). Table 1 shows some studies in which the DEA approach was carried out in Health-Agricultural-Public systems for optimization.

**TABLE 1:** The application of the data envelopment analysis (DEA) approach in health-agricultural-public systems.

Surveyed research	Studied area	Sector	Optimization by DEA	Resource management	Effective factors
Jia and Yuan (12)	China	Health system	Yes	Yes	No
Rosić et al. (13)	Serbia	Public	Yes	No	No
Zhou et al. (14)	China (Jiangsu)	Health system	Yes	No	No
Sun and Luo (15)	China	Health system	Yes	No	No
Li et al. (16)	China	Agriculture	Yes	No	Yes
Nabavi-Pelesaraei et al. (17)	Iran	Agriculture	Yes	Yes	No
Li et al. (18)	China	Agriculture	Yes	No	No
Li et al. (19)	China	Environment	Yes	No	No
Li et al. (20)	China	Agriculture	Yes	No	No
Rego et al. (21)	Brazil	Agriculture	Yes	Yes	No
Grados and Schrevens (22)	Peru	Agriculture	Yes	No	No
Nabavi-Pelesaraei et al. (23)	Iran	Agriculture	Yes	Yes	No
Zhang and Zhou (24)	China	Industry	Yes	No	No

Considering the urgent need to increase human health, this paper is aimed to:

➢ Develop a novel DEA and regression model to measure food consumption efficiency.

➢ Determining the best combination of food groups for nutrient security using current food resources consumed by Iranian households.

➢ Identifying factors associated with nutrient efficiency by Tobit model.

➢ Proposing a method for depicting the geographical plot of nutrient efficiency for both rural and urban areas.

## Materials and Methods

### Sample Description

Data and information are gathered from the Statistical Centre of Iran (SCI), which is responsible for the collection of the Iranian household expenditures and income annually through a questionnaire with proven reliability and validity. The SCI survey includes the consumption of 167 food ingredients within a household. The respondent is either the breadwinner or a member aware of the consumption of all household members, able to answer all questions on behalf of the breadwinner (2).

We used the household data to measure nutrient intake. Inputs and outputs for each province were calculated by averaging household data in each province. Information of about 30,000 rural and urban households was gathered in 2016. All 167 food ingredients were divided into 14 food groups to calculate nutrient content consumed by households (25) in 316 townships. The coefficients of the nutrient content of food ingredients extracted from the National Nutrition and Food Technology Research Institute (NNFTRI) were used.

### Nutrient Intake Evaluation

Nutrient intake, such as calories, protein, calcium, iron, vitamin A, and vitamin C, was used to identify households’ nutrient status. Assuming a linear function, adult nutrient intake can be calculated by Eqs. 1, 2:


(1) $$y_{i}=\sum_{j=1}^{n=k}\beta_{j}\,Z_{i\,j}+\varepsilon_{i}$$

**FIGURE 1:**
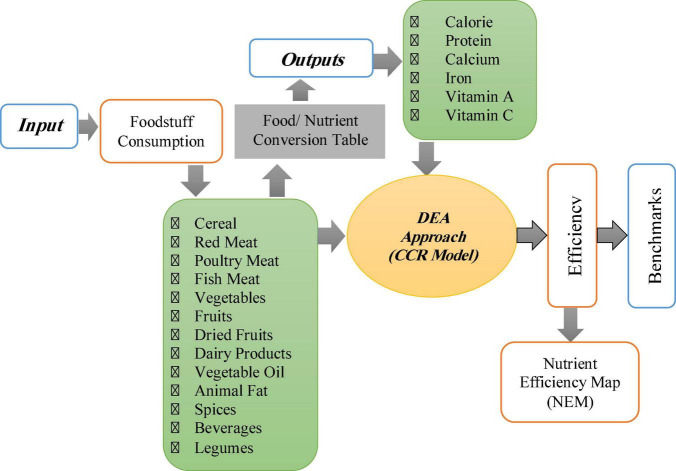
The conceptual framework of the extraction of nutrient intake efficiency for Iranian households.

Where *y*_*i*_ is the level of nutrient intake of each household member in a day; *Z*_*ij*_ is the amount of *i*-th food group consumed by the *j*-th person in a day, and *B*_*j*_ is the nutrient contents of *j*-th foodstuffs. By dividing this matrix into the average number of household members (26) in each province, the matrix of nutrient intake for a person per year was calculated. Optimization of food available for consumption was calculated by DEA as shown in Figure 1.

### Data Envelopment Analysis

There are two main DEA models that refer to constant and variable returns to scale (CRS and VRS). We will be using a CRS model, since increasing consumption will increase output (here is nutrient intake) with the same ratio (27). In respect to orientation, the aim of this assessment is on reducing unnecessary food consumption, and hence input orientation was assumed to minimize the food consumption and identify the level of food overconsumption. Consider a set of n DMUs, where DMUj has a consumption plan (X_*j*_, Y_*j*_) with *X*_*j*_ = (*x*_1*j*_,*x*_2*j*_,…,*x*_*mj*_) inputs (food groups) and *Y*_*j*_ = (*y*_1*j*_,*y*_2*j*_,…,*y*_*sj*_) outputs (nutrients intake). Suppose that *x*_*ij*_(*i* = 1,2,…,*m*) is the quantity of food group i consumed by household j (DMUj) and *y*_*rj*_(*r* = 1,2,…,*s*)is the quantity of nutrient intake by household j (DMUj) (28). Therefore, the following DEA model can be used to measure nutrition intake efficiency.


$$\max\theta +\varepsilon \,\left(\sum_{i=1}^{m}S_{i}^{-}+\sum_{r=1}^{s}S_{r}^{+}\right)$$

S⁢ubject⁢to

(2) $$\sum_{\text{j}=1}^{\text{n}}\lambda_{\text{j}}\,x_{i\,j}+S_{i}^{-}=\theta^{{}^{*}}\,x_{i\,0}\;i=1,2,\ldots ,m$$

$$\sum_{\text{j}=1}^{\text{n}}\lambda_{\text{j}}\,y_{r\,j}-S_{r}^{+}=y_{r\,0}\;r=1,2,\ldots ,s$$

$$\lambda_{\text{j}}\,\geq \,0$$

ε≥0

Where the optimal θ* represents the efficiency in the input-oriented model; $S_{i}^{-},\,S_{r}^{+}$ are the slacks of food ingredients and macro and micronutrients, respectively; n represents the number of households; *j* = 1,2,…, 30,000 represents indexes for households; m represents the number of food ingredients; *i* = 1,2,…, m represents an index for food ingredients; s represents the number of macro- and micronutrients, r = 1, 2, …, s represents an index for nutrient intake, and λ is called the intensity vector. Household is efficient if and only if θ* = 1 and $S_{i}^{-}=S_{r}^{+}=0$for all i and r. Household is weakly efficient if θ* = 1, and at least one of the slack is non-zero (i.e.,$S_{i}^{-}\neq 0$ or $S_{i}^{+}\neq 0$ for some i or r).

### Associated Factors With Nutrient Efficiency: A Generalized Linear Model

Table 2 shows variables that are chosen as potential determinants of nutrient efficiency based on the related literature review.

**TABLE 2:** Definition of variables used in GLM.

Variable		Definition	Average		Percent	
			Rural	Urban	Rural (=1)	Urban (=1)
HH-Social	HH-Age	Age of household head in townships	50.9	49.6	–	–
	HH-Educated	The education of households head (educated = 1, otherwise = 0)	–	–	38.5	23.4
	HH-Sex	Sex of household head (man = 1, otherwise = 0)	–	–	88.4	89.3
	HH-Marital	Marital status of household head (HH who is married = 1 and otherwise = 0)	–	–	87.6	87.7
	N	Household size	3.6	3.4	–	–
	N-Student	The number of students in household	0.9	1.0	–	–
	N-Educated	The number of educated in household	2.8	3.1	–	–
	N-Employee	The number of employees in household	1.3	1.1	–	–
	HH-Employee	The employee status of household head (employing = 1)	–	–	95.2	95.0
	HH-Keeping	1 if female headed household is housewife, 0 if otherwise	–	–	98.9	99.1
HH-Economical	SH-Agriculture	Agricultural income share of households incomes	–	–	13.5	3.0
	SH-Food	Household food share of total expenditure	42.6	33.8	–	–
	DDS*^a^*	Food diversity (percentage of consumption goods of total 167 goods)	9.1	8.2	–	–
HH-Assets	Home	Household some status (private home = 1)	–	–	93.7	82.2
	S-Home	Home area (m^2^)	89.1	101.9	–	–
	Machine	Household machine status (private machine = 1)	–	–	23.7	36.3
HH-Cultural	Azeri language	Township in ethnic Azeri language = 1, otherwise = 0	–	–	14.2	14.5
HH-Geographical	Capital city	Capital cities = 1, otherwise = 0	–	–	10	9.7
	Sea-Cities	Townships on the edge of the sea	–	–	10.6	10
	Tropical-Cities	Townships in the tropical regions = 1, otherwise = 0	–	–	14.8	14.8

*^a^DDS was calculated through HDDS (number of different food groups consumed over a given reference period) (25). Firstly, the HDDS variable is calculated for each household. The value of this variable ranges between 0 and 12. Secondly, the average HDDS indicator is calculated for the sample population (29).*

In the second stage of the analysis, we used a censored regression (Tobit) to assess associated factors with nutrient efficiency:


(3) $$\theta^{i}=\sum_{i=1}^{R}\beta_{i}\,W_{i}+\varepsilon_{i}$$

Where θ*^i^* is the efficiency calculated from solving the mathematical programming problem 2 above and used as a dependent variable and *W* is a (*R*×1) vector of independent variables related to attributes of the households in the townships. The dependent variable has two values as follows:


(4) $$\theta^{i}=\left\{ \begin{array}{c} \theta^{*}\;\mathrm{if}\,\theta^{*}>0\\ 0\;\mathrm{if}\,\theta^{*}\leq 0 \end{array}\right.$$

Where θ* is a latent variable. Table 2 summarizes the statistical description of all factors used in this study.

## Results

### Nutrient Intake Efficiency Analysis

In the first step, we calculated the average nutrition intake of an individual in the urban and rural areas. There is a significant difference among all nutrient intakes between rural and urban areas. As can be seen in Table 3, the nutrient intake of an individual in the rural areas was greater than in urban areas, except for vitamin A and C. The calorie intake of an individual is 3,615/cal/per person in rural areas, while the intake of this macronutrient is 3,280/cal/per person in urban areas.

**TABLE 3:** The calculated nutrient intake in rural and urban areas in Iran.

Nutrition	Rural areas				Urban areas				Difference
	Average	S.E.	Max	Min	Average	S.E.	Max	Min	
Calorie (cal)	3615	258.3	5178	1859	3280	239.3	5455	1553	−6.92***
Protein (gr)	94	18.05	125	55	82	15.06	116	49	−10.7***
Calcium (milli-gr)	747	145.4	1022	422	689	134.5	1013	412	−8.02***
Iron (milli-gr)	23	4.58	33	14	21	4.24	31	13	−6.74***
Vitamin A (micro-gr)	1558	403.9	2606	675	1718	402.4	2412	799	4.84***
Vitamin C (milli-gr)	96	26.13	675	54	103	25.18	153	49	2.87***

***Indicates significance at 1% level. Cal, calorie; Gr, gram; mili-gr, milligram; and micro-gr, microgram.

Intake of all macro- and micronutrients is really important to be healthy. Table 4 shows that the average foodstuff consumption of rural households is greater than in urban areas. According to the results, the average consumption of all food groups in the rural area was greater than in the urban area. As Table 4 shows, the most important part of the difference between calorie intake between individuals in rural and urban areas is related to the consumption of cereal.

**TABLE 4:** Descriptive statistical analysis of measured food ingredients by the households in urban and rural areas in Iran.

Food groups	Urban				Rural				Difference
	Average (kg/month)	S.E.	Max	Min	Average (kg/month)	S.E.	Max	Min	
Cereal	50	14.87	91	29	62	18	102	33	−9.4***
Vegetable	5.5	3.07	13	1.7	9	4.69	21	1.6	−7.2***
Fruits	4.2	1.34	8	2.1	9	3.07	19	4	−4.6***
Dried fruits	1.8	0.83	4	0.63	1.7	0.99	5	0.56	0.32
Dairy products	13	3.32	19	6.7	16	4.58	25	7	−9.6***
Legumes	0.47	0.33	2	0.08	0.6	0.39	2	0.08	−3.1***
Fish meat	2.8	0.41	4	1.8	3	0.49	4	1.7	−4.5***
Poultry meats	22	4.68	33	13	25	7.62	45	13	−5***
Red meats	2.4	1.12	6	0.69	3	1.30	6	0.44	−0.86
Vegetable oils	16	3.74	27	9.5	18	4.90	30	9	−3.9***
Animal fats	2.9	0.89	6	1.4	3	0.84	6	1.6	−5.6***
Sweets	4.7	1.10	7	2.9	5	1.64	9	2.3	−6.3***
Spices	4.4	1.95	10	2.1	5	2.26	11	2.1	−4.3***
Beverage	0.46	0.26	2	0.15	0.44	0.17	1	0.11	−2.3***

***Indicates significance at 1% level.

The nutrient efficiency of all townships was estimated using DEA. Townships with a score of 100 had the best nutrient consumption efficiency. Then, the provincial score was calculated by averaging the efficiency score of townships within each province. Table 5 shows the average efficiency score of each province. According to the DEA calculated scores in the different townships in all provinces, the average efficiency in Alborz, East Azerbaijan, Chaharmahal and Bakhtiari, Kerman, Khuzestan, Qazvin, Tehran, Yazd, and Zanjan is different between rural and urban areas. This result may be related to the optimized food consumption of the households or the work of rural residents, which requires more calories than urban work.

**TABLE 5:** Townships with full efficiency in the rural and urban areas in Iran [identified by the calculated data envelopment analysis (DEA) score in the study].

Province	Rural	Urban
**Alborz**		
**Ardabil**		
Bushehr	Dashti and Deyr	
East Azerbaijan		Malekan and Varzaghan
Fars	Abadeh, Mehr, and Neyriz	
Hamadan		Ranen
Hormozgan	Abu Musa, Parsian, and Minab	Bastak and Khamir
Ilam		Darreh Shahr
Isfahan	Shahreza and Fereydunshahr	Dehaghan, Fereydan, and Fereydunshahr
Kermanshah	Sahneh and Gilan-e-Gharb	Paveh and Gilan-e-Gharb
Khuzestan	Abadan, Bandar-e-Mahshahr, Khoramshahr, Shadegan, Shushtar, and Masjed Soleyman	Andimeshk, Ramshir, Shush, and Shushtar
Kurdistan	Bijar	
Mazandaran	Savadkuh	Juybar, Savadkuh, Galugah, and Noshahr
Semnan		
Sistan and Baluchestan	Nikshahr	Zahak and Konarak
South Khorasan		Darmian
Tehran	Eslamshahr	
West Azerbaijan	Bukan and Takab	Oshnavieh
Zanjan	Ijrood	Ijrood and Tarom

**FIGURE 2:**
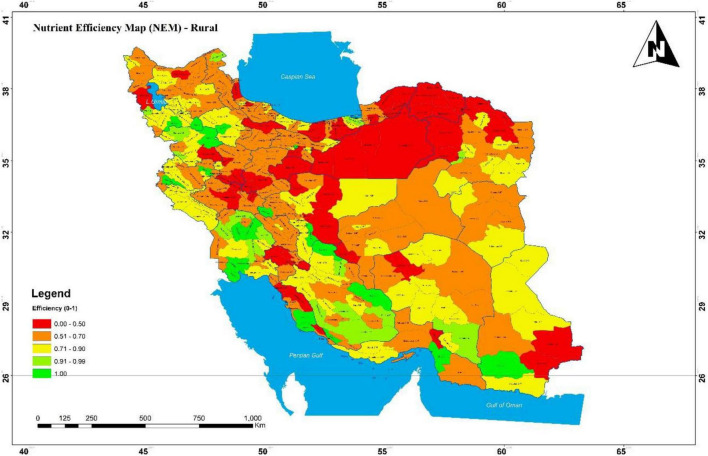
Nutrient intake efficiency map (NIEM) in the rural area in Iran, 2016.

**FIGURE 3:**
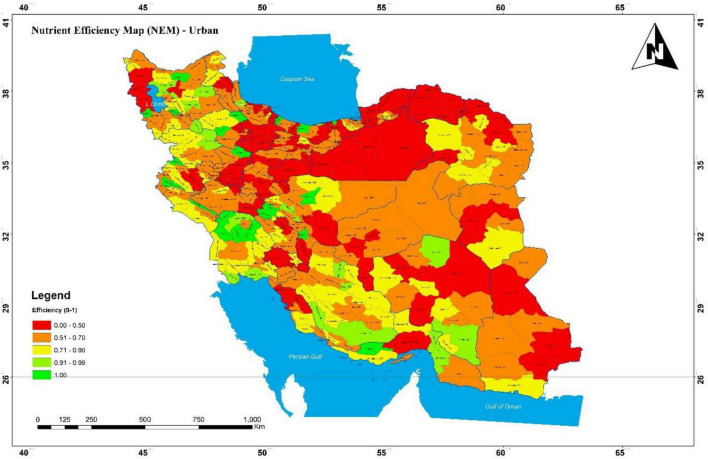
Nutrient intake efficiency map in the urban area in Iran, 2016.

Rural and urban nutrient efficiency was compared by the province in the form of a map (see NEM in Figures 2, 3).

**FIGURE 4:**
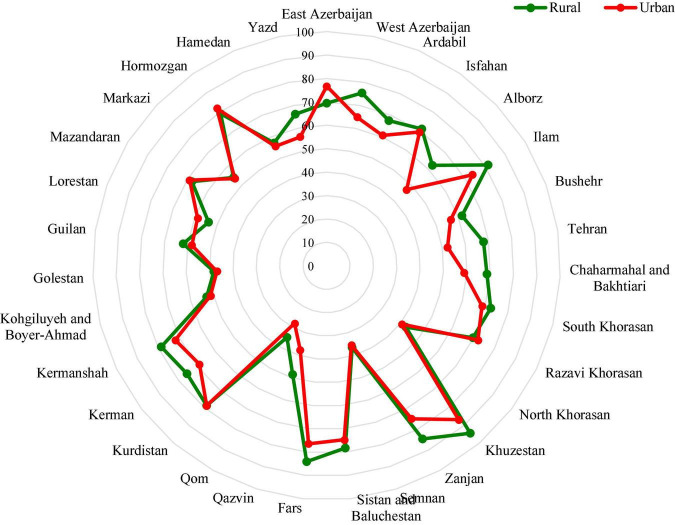
Comparison of the average nutrient efficiency in the provinces in both rural and urban areas.

This map has been developed using GIS software, encompassing a broad range of applications using a combination of digital maps and geo-referenced data. Many rural townships had efficiency scores of less than 70%, which are even lower in the Northeast. North, East, and Northeast townships had low urban nutritional efficiency, even lower than their rural areas. In central Iran, rural areas were more nutrient efficient than urban areas. Khuzestan had the highest efficiency in both rural and urban areas, followed by Fars, while Qom was the lowest. Comparison of two areas in Figure 4 demonstrates that the average efficiency in the rural areas is higher than in urban areas among most provinces in Iran. As can be seen in Figure 4, the greatest gap in nutrient efficiency between rural and urban areas is in Alborz province.

The results indicated that a better-balanced food group’s consumption was found in rural as compared to urban areas.

### Associated Factors With Nutrient Intake Efficiency

Factors associated with nutrient intake efficiency were determined in both rural and urban areas. Table 6 includes the estimated coefficients from the Tobit along with the z-statistics and shows differences between the two areas. Age of household head was positively and significantly associated with nutrient efficiency in both rural and urban areas. Households whose heads were educated had better nutrient efficiency than other households. Male-headed households had a lower nutrient efficiency than female-headed households in urban areas, while the gender of the household head had no impact on nutrient efficiency in rural areas. Household size was directly and positively associated with nutrient efficiency only in rural areas. Dietary diversity was positively and significantly associated with the nutrient efficiency in both rural and urban areas, with the association stronger in the rural areas than in urban areas. Ethnicity was significantly and directly associated with nutrient efficiency. Azeri areas, the densely ethnic population in the northwest of the country, had a higher nutrient efficiency as compared to other ethnicities. Tropical areas, such as Ilam, Khuzestan, Bushehr, and Sistan and Baluchistan, had higher nutrient efficiency than cold regions. Eventually, some factors that include capital cities, the share of agricultural income, and married head of household were inversely and significantly associated with nutrient efficiency in both rural and urban areas.

**TABLE 6:** Effective factors on nutrient inefficiency in the rural and urban areas.

Item		Rural		Urban	
		Coefficient	Z-statistics	Coefficient	Z-statistics
***A. Variable***					
HH-Social	HH-Age	−0.0080***	–2.46	−0.0023***	–2.81
	HH-Educated	−0.0031*	–1.96	−0.0027**	–2.06
	HH-Sex	0.0032	1.39	0.0013*	1.78
	HH-Married	0.0037*	1.62	0.0033*	1.73
	HH-Employee	0.0006	0.29	−0.0038**	–2.25
	HH-Keeping	0.0049**	2.24	−0.0082*	–1.74
	N	−0.1739***	–2.98	–0.0373	–0.61
	N-Student	–0.0107	–0.19	−0.0180**	–2.10
	N-Educated	0.1071*	1.68	0.0854***	4.20
	N-Employee	−0.0551**	–1.90	−0.1838***	–3.87
HH-Economical	SH-Agriculture	0.0031**	2.10	0.0034***	5.31
	SH-Food	−0.0063***	–3.44	−0.0096***	–4.47
	DDS	−0.0117***	–5.84	−0.0057***	–2.93
HH-Assets	Home	0.0029***	2.72	0.0006	0.06
	S-Home	0.0001	0.08	−0.0007**	–2.31
	Machine	−0.0012*	–1.71	0.0022***	3.02
HH-Cultural	Azeri language	−0.0559**	–1.88	−0.0869***	–3.17
HH-Geographical	Capital city	0.1342***	4.61	0.1868***	5.77
	Sea-Cities	0.0162	0.52	0.0461***	3.18
	Tropical-Cities	−0.0730**	–2.30	−0.0588*	–1.74
*B. Constant*	–	0.8692***	2.46	0.8014**	2.55

*, **, *** Indicate significance at 1, 5, and 10% level, respectively.

## Discussion

Our work established the value of FOC and Optimal Nutrient Intake Efficiency (ONIE) within optimizing frameworks for studying nutrient security. Empirically, the utility of using a combination of the DEA and Tobit model is vindicated. A large data set to derive policy-relevant results can be used within our models.

Nutritional education increases knowledge of food combinations (30, 31), and as a result, household nutrient efficiency increases. In this paper, an innovative approach was developed to identify the association between food combinations and nutrient efficiency in urban-rural areas in Iran using a two-stage approach. According to the descriptive results, the nutrient intake of Iranian households in rural areas was greater than in urban areas. This result may be associated with higher agricultural and animal production by rural households (32, 33). Rural households can easily access adequate food ingredients, especially raw ingredients, which help them to access needed nutrients without extra payment.

In the first phase, the nutrient efficiency of households was calculated using the DEA approaches. The results showed that the combination of food ingredients was not utterly optimal in all provinces in both rural and urban areas. Rural households had 30% nutrient inefficiency and urban households had 35% inefficiency in food group consumption. Nutrient efficiency was greater in rural areas than the urban areas, and generally, the availability of healthier foodstuff combinations in the rural areas is better than in urban areas. The provinces of Qom and Semnan had the lowest efficiency in both rural and urban areas. The highest rural and urban inefficiency scores were in the provinces of Fars, Khuzestan, and Hormozgan. These high scores may be associated with a high level of education in these provinces contributing to choosing the best combination of food ingredients.

Most of the cities face nutrient inefficiency, especially in the northeast of the country. Households could increase their nutrient intake by ameliorating food group combinations. Food combinations in high-efficiency areas could be used as a guideline to improve nutrient status in low-efficiency areas. For example, food ingredient combination in Khuzestan province, a better location in terms of nutrient efficiency, can be monitored and addressed by the adjacent provinces for improving households’ nutrient status.

Benchmarks for each province to reach ONIE provide the targets for Iranian households to balance expenditures and consumptions for health protection. For better nutrient efficiency in households in Tehran, a densely populated capital city, households should decrease cereal consumption by 30% per month. To optimize foodstuff consumption (receiving the current nutrient intake) and to reduce households’ expenditure in Tehran in comparison with other provinces where households have the better ONIE, households should decrease, per person, the consumption of all food groups. The dietary pattern in some provinces was sub-optimal.

The nutrient status of Iranian households can be improved by decreasing their food expenditure without changing their nutrient intake. As the results confirmed, most of the Iranian households were engaged in an overconsumption pattern in all provinces, low in some provinces and high in others. This status can be achievable by controlling the overconsumption pattern and allocating the extra expenditure to high-quality nutrients. That could allow the country to save strategic food ingredients, and, eventually, decrease the amount some households spend on food. This could be important for Iran, as one of the biggest importers of food ingredients worldwide.

The direct association between the age of the head of the household and nutrient efficiency suggests that older heads have more experience in combining food ingredients and also their knowledge and capability of food combinations lead to increasing nutrient efficiency (34, 35). Some studies contended that elder heads have an adequate ability to food money management along with experience better dietary quality (36).

Higher education of household heads is associated with increasing the nutrient efficiency of food consumption, and so food overconsumption is reduced. This result contradicts a study in Suriname which found that both adequate and excessive consumption were increased with the higher degree of urbanization, level of education, and income, with the exception of the highest categories of education and income (4), because the level of Suriname people’s income is generally low. Therefore, a slight increase in income level or education level will lead to extending the overconsumption pattern due to increasing the level of people’s expectations, especially in the low and middle-income groups.

Female-headed households had greater nutrient efficiency in urban areas. A female head may have more experiential knowledge of combining different foods, as they play an essential role in the nutrition of children and other members (37). Some studies found that male-headed households have higher nutrient security (38, 39). These results are attributable to the lower incomes and lower access to resources of female-headed households. Once the confounding causal influence of income and economic status is controlled, female-headed households have higher nutritional efficiency.

Household size had a direct and significant association with nutrient efficiency in rural areas. Due to the larger size of rural households, they endeavor to use their food ingredients appropriately and completely, and therefore, the amount of food overconsumption is lower than in smaller households. The association between household size and food consumption has been found to be positive in some studies (32, 34) or negative in others (33, 40).

The share of income from agriculture is inversely associated with nutrient efficiency. When rural households access easily adequate food ingredients through agricultural production, they overconsume their resources. In fact, rural dwellers are confident that they always access the needed raw ingredients (41) and do not intend to control their consumption.

An increase in the number of different food groups consumed provides a quantifiable measure of improved household food access and nutrient consumption. In general, any increase in household dietary diversity reflects an improvement of the household’s diet quality (25). An increase in the number of consumed food groups will lead to increased nutrient intake efficiency, eventually increasing nutrient security. In contrast with our findings, some studies contended that living in urban areas is associated with higher dietary diversity that includes higher fruit and vegetable intake, in low- and middle-income countries.

Azeri people had more nutrient efficiency than other ethnicities in Iran. This result suggested that this population has a high ability to combine food ingredients in an optimized level for achieving nutrient efficiency as compared to other ethnicities. As a geographical pattern of food consumption demonstrated, people of Azeri ethnicity, living in the northwest of Iran, can prepare different kinds of food through distinct food ingredients that lead to a decrease in food overconsumption.

The geographical location of a province was significantly associated with nutrient efficiency. Living in the capital province led to the decrease of the NIE in both rural and urban areas. Household income in Tehran is higher than other locations in Iran, so there is no felt need to optimize food ingredients. Marques et al. (42) found that geographical location is a key factor in controlling food overconsumption and recycling systems. On the other hand, a study contended that, due to the overconsumption pattern, the rate of overweight and obesity in Tehran province was more than the country rate. About 42.7% of all participants in that study had overweight and 45% had obesity.

Nutrient efficiency is directly associated with the type of knowledge of people in different locations. According to the food culture of people in seaside cities, the NIE of households who are living in these cities is lower than those who are living in other locations. The abundance of food, plentiful local and traditional foods, diet diversity, and access to adequate food ingredients may be the main reasons for this negative association in these seaside cities.

Ultimately, some countries, especially developing countries, face prevailing food insecurity due to the lack of accessibility to adequate resources and enough foods (43). The results of such studies can be used to determine the accurate consumption of food resources, prevent from the destruction of more resources for gaining more foods, and finally contribute to people saving their incomes and reducing their expenditure.

As some international reports showed, the best combination of food ingredients is really important in many African countries, because most of these countries face large numbers of hungry and food insecure population (44). Many poor African countries could better address the prevalence of undernourishment by using their available limited budgets optimally according to the methodology presented in our paper. Understanding socio-economic determinants of food efficiency is a key component of developing policies that will allow a country to maintain its resources. A comprehensive study of nutrient efficiency through sophisticated techniques will help policymakers to find the lowest cost approaches for determining the best food baskets. This study has implications for a policy most immediately within Iran. More broadly, the results could be generalizable for other MENA countries and be applicable with further country-specific empirical work to other economies in the developing world that have income levels and degree of urbanization and geographical distribution similar to those of Iran.

Although consumers in developed and high-income countries have been identified as the biggest single contributor to food overconsumption and waste (45–47), our study suggested that this claim is also the case in developing countries. Further studies are needed to investigate the NIE and calculate better ways to optimize food intake in different regions, cities, and countries. Future studies should examine how households in different provinces with a high nutrient efficiency allocate their food ingredients to food preparation. That information could aid the formulation of new policies to promote higher nutrient efficiency and control the consumption of strategic and staple food ingredients.

## Conclusion

The food patterns in the provinces with a high score of nutrient efficiency could be used as a guideline for other parts of the country by the government. But households also need monetary resources to access healthy foods, which must be available at reasonable prices in local markets. According to the efficiency map drawn from our empirical analysis, policies should be directed to specific geographical area-based households, especially in the Northeast, where the households face the lowest level of nutrient efficiency due to inappropriate food patterns. Socio-economic and cultural factors are associated with nutrient intake efficiency. Educating households regarding the correct combination of food ingredients to increase nutrient knowledge is necessary. The diffusion of such education could be done by broadcasting TV commercials for health-promoting dietary patterns and optimal combinations of culturally appropriate foods. Investment in primary education that includes nutrition education may increase nutrient efficiency in the long term. Moreover, the association of geographical factors can be considered an important dimension in prescribing a health policy, because being in tropical cities is associated with greater nutrient efficiency. The story in the capital province and coastal cities is completely different. Designing different training packages of food combinations, focusing on the exact reasons for the low level of optimal nutrient intake efficiency in such areas, and constructing the specified number of urban zones based on the similar nutrient intake efficiency (or close efficiency) may help policy makers and planners to save national and urban resources without declining people’s nutrient level.

This study has several limitations as other studies confront. Verification of the results is directly associated with the accuracy of filling out the questionnaires. Another limitation of this study is the need to disaggregate the large data set that included all counties and provinces in Iran. The use of the food composition table suggested by the NNFTRI could be a limitation because some of the commodities used by Iranians are not included in the table, especially imported commodities because the nutrient contents of these commodities are different.

## Data Availability Statement

The datasets presented in this study can be found in online repositories. The names of the repository/repositories and accession number(s) can be found below: https://www.amar.org.ir.

## Author Contributions

MP-C: supervision, formal analysis, methodology, and writing–original draft preparation. CF: conceptualization, validation, and reviewing and editing. AE: writing methodological advise, interpretation, and reviewing and editing. All authors contributed to the article and approved the submitted version.

## Conflict of Interest

The authors declare that the research was conducted in the absence of any commercial or financial relationships that could be construed as a potential conflict of interest.

## Publisher’s Note

All claims expressed in this article are solely those of the authors and do not necessarily represent those of their affiliated organizations, or those of the publisher, the editors and the reviewers. Any product that may be evaluated in this article, or claim that may be made by its manufacturer, is not guaranteed or endorsed by the publisher.
